# Seroprevalence of Sindbis virus and associated risk factors in northern Sweden

**DOI:** 10.1017/S0950268813002239

**Published:** 2013-09-13

**Authors:** C. AHLM, M. ELIASSON, O. VAPALAHTI, M. EVANDER

**Affiliations:** 1 Department of Clinical Microbiology, Infectious Diseases, Umeå University, Umeå, Sweden; 2 Department of Public Health and Clinical Medicine, Sunderby Research Unit, Umeå University, Umeå, Sweden; 3 Department of Virology, Haartman Institute and Department of Veterinary Biosciences, University of Helsinki, Finland; 4 Department of Virology and Immunology, Helsinki University Central Hospital Laboratory, Finland; 5 Department of Clinical Microbiology, Virology, Umeå University, Umeå, Sweden

**Keywords:** Arboviruses, epidemiology, risk assessment, Sindbis virus, zoonoses

## Abstract

Mosquito-borne Sindbis virus (SINV) cause disease characterized by rash, fever and arthritis which often leads to long-lasting arthralgia. To determine the seroprevalence of SINV and associated risk factors in northern Sweden, a randomly selected population aged between 25 and 74 years were invited to join the MONICA study. Serum from 1611 samples were analysed for specific IgG antibodies. Overall, 2·9% had IgG against SINV. More men (3·7%) than women (2·0%) were SINV seropositive (*P* = 0·047) and it was more common in subjects with a lower educational level (*P* = 0·013) and living in small, rural communities (*P* < 0·001). Seropositivity was associated with higher waist circumference (*P* = 0·1), elevated diastolic blood pressure (*P* = 0·037), and history of a previous stroke (*P* = 0·011). In a multiple logistic regression analysis, adjusting for known risk factors for stroke, seropositivity for SINV was an independent predictor of having had a stroke (odds ratio 4·3, 95% confidence interval 1·4–13·0, *P* = 0·011).

## INTRODUCTION

Mosquito borne alphaviruses within the Togaviridae family, including Sindbis virus (SINV), Chikungunya virus (CHIKV) and Ross River virus (RRV), are a major cause of debilitating arthritic disease worldwide [1]. They are all transmitted to humans from their natural reservoirs by mosquito bites and for SINV, migratory and/or resident birds serve as reservoirs [2]. SINV antibodies are found in birds, wildlife and human populations in many parts of the world, but intriguingly human disease is predominantly appearing in northern Europe. Genetic risk factors could play a role and for SINV it was recently shown that HLA-DRB1*01 was associated with clinically manifest SINV infection [3]. In Sweden, SINV infection in humans is called Ockelbo disease, in Finland Pogosta disease and in Russia Karelian fever [4, 5]. However, reports of the disease are lacking from most other European countries. In most of Europe, SINV or SINV antibodies have been detected [6, 7] raising the possibility of a potentially important but neglected disease.

Ockelbo disease is not a mandatory notifiable disease in Sweden, thus the exact number of cases is not known. In a seroepidemiological study from human sera collected during 1981–1987 the antibody prevalence to SINV was 3·6% in central Sweden with a decreasing seroprevalence rate south and north of this region. In the two northernmost counties only 0·1–1·0% of the population had SINV antibodies [8]. SINV infection is the most commonly diagnosed arbovirus infection in neighbouring Finland with an estimated seroprevalence of 5·2% [2]. In Finland serologically diagnosed SINV infection is mandatory notifiable and Finland has previously experienced recurrent outbreaks at roughly 7-year intervals [9]. In Sweden a similar pattern has been described [10].

Ockelbo disease is characterized by rash, headache, fever, viraemia, arthritis, and myalgia [3, 11]. Even though SINV viraemia is usually short-lived, arthralgia and myalgia can persist for several months and even years following infection [12, 13]. In one study from Finland, 25% of patients with SINV infection had persisting symptoms 3 years after acute disease, of which 14% could be verified by a rheumatologist [14]. Viral RNA from another alphavirus, RRV, has been detected in synovial fluid from an affected joint, which may be a possible explanation for arthritis years after the acute infection [15]. Viral arthritis could be underestimated as a contributory factor in different rheumatic diseases and very few studies have approached this question although prolonged joint manifestations are a considerable public health problem in areas where SINV is present [2, 13].

Vector-borne viral diseases have shown a propensity for emergence or re-emergence and recently southern Europe experienced an alphavirus outbreak when CHIKV emerged in Italy [16]. Similarly, SINV infections have increased in Finland since the 1970s [2, 9, 17] and it could also be the case for neighbouring Sweden. The present study was motivated by the lack of current information concerning SINV infection in Sweden and a possible involvement in joint-related illnesses, which constitutes a common public health problem.

For that reason we aimed to determine the current SINV seroprevalence and epidemiological factors associated with a previous SINV infection using a unique and randomly selected population-based cohort from northern Sweden.

## METHODS

### Survey participants

This study used data from the northern Sweden MONICA study [18]. In 2009, a population-based survey was conducted in the two most northern counties of Sweden (target population 312 000). Subjects aged 25–74 years were randomly selected from population registers and stratified for age and gender. Details of sampling and selection have been presented elsewhere [19]. Data on non-participants have been published [18].

### Measurement procedures

The highest attained educational level was classified as primary school or secondary school (9–12 years of school) and university studies. The participants were asked to describe their main occupation and agricultural work was compared to all other occupations. In addition, it was asked whether the participants were owners of farmland or forests.

The prevalence of regular smokers, diabetes, treated hypertension, previous myocardial infarction, arthralgia, myalgia and calf pain on exertion were based on the MONICA questionnaire (exact wording of the questions are given in the online Appendix). The prevalence of previous stroke was based on the question ‘Have you had a stroke (bleeding in the brain or clot in the brain)? If yes, give the name of the hospital and the year’.

Blood pressure was measured twice, after a 5-min rest, in a sitting position. Subjects were weighed on an electronic scale and hip and waist circumferences were measured.

Blood samples were drawn after at least a 4-h fast. Total cholesterol was determined by a dry chemistry method (Vitros 950, Kodak Echtachem, USA) [20]. Creatinine was measured by a dry chemistry method on a Vitros 5.1 instrument (Ortho Clinical Diagnostics, USA).

### SINV serology

Specific IgG against SINV was measured by using an enzyme immunoassay (EIA) as previously described [21]. Briefly, the SINV antigen was water-sonicated on ice for 4 × 30 s and was box-titrated to determine the optimal antigen dilution. Diluted sera (1/420) were incubated at 4°C overnight on antigen-coated wells, washed four times with PBS-T, again incubated for 60 min at 37°C with 100 *μ*l goat anti-human IgG alkaline phosphate conjugate (Invitrogen Corporation, USA, lot: 722339A) (1:6000). Substrate (*p*-nitrophenyl phosphate disodium; Sigma Diagnostics, USA) was added and incubated for 30 min at 37°C and the optical density (OD) was recorded at 405 nm. The mean OD of eight blank wells (containing PBS-T and 1% milk) was subtracted from each result. The cut-off in SINV IgG EIA was determined by analysing 32 sera, previously confirmed SINV negative by immunofluorescence assay. The assay cut-off was preliminarily determined by counting the mean OD value of these SINV negative samples +3 standard deviations. All sera were first analysed once and those with an OD above cut-off were analysed again in duplicate. The SINV antibody-positive results from the EIA were verified using a SINV-specific haemagglutination inhibition (HI) test [21].

### Ethical approval

The study was approved by the Research Ethics Committee of Umeå University; all subjects gave informed consent to participate in the MONICA project.

### Statistics

The *χ*^2^ test and Student's *t* test were used for group comparisons. Multiple stepwise logistic regression was used to analyse possible independent lifestyle and demographic predictors of SINV seropositivity. The stepwise logistic regression with prevalent stroke as dependent variable included SINV seroprevalence and variables associated with it in univariate analysis. Moreover, known traditional risk factors for stroke were included (education, smoking, waist circumference, diastolic blood pressure, smoking). Outcome is presented by odds ratios (ORs) with corresponding 95% confidence intervals (CIs). Statistical analyses were performed using IBM SPSS 20 (USA).

## RESULTS

In total, 2500 subjects were invited to participate of which 1729 (69·2%) did so. Lowest participation rates were found in the 25–34 years age group. In the oldest age group, 65–74 years, participation was 80% and 74%, in men and women, respectively.

**Fig. 1:**
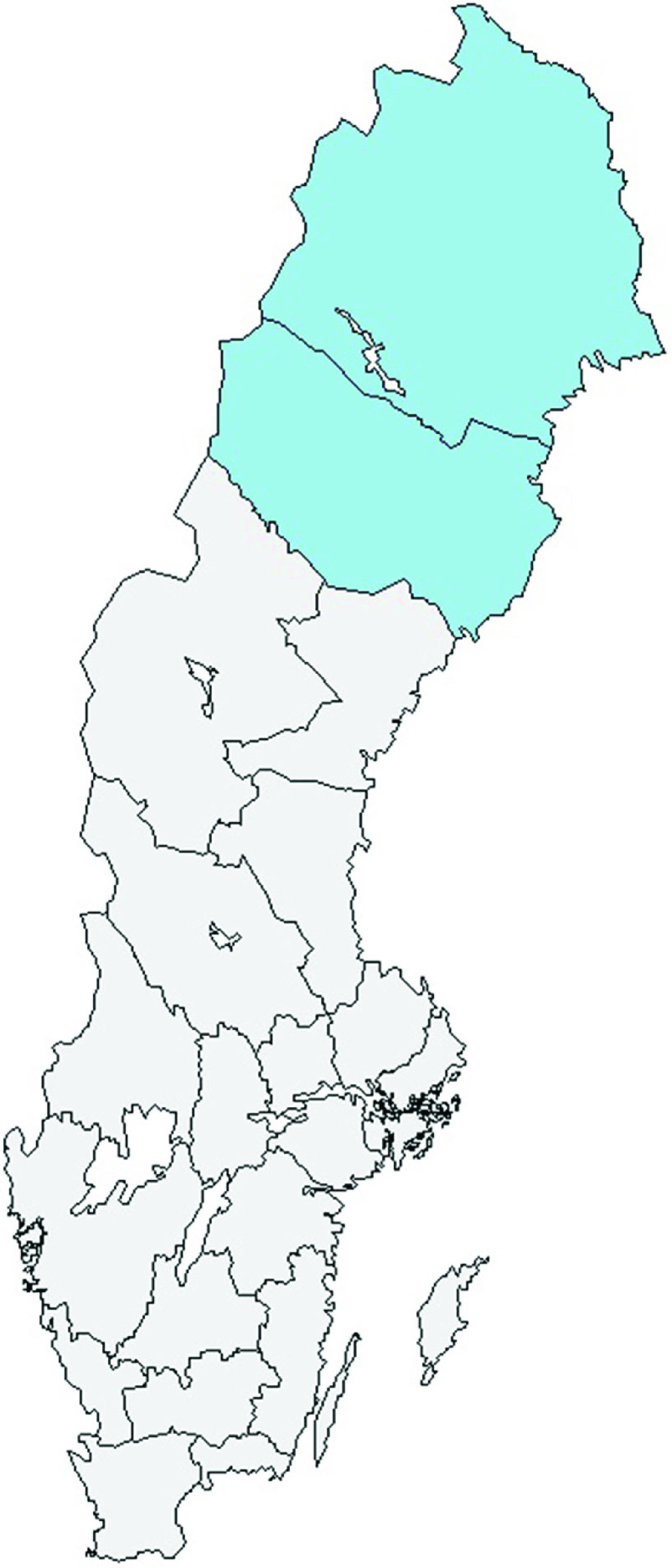
[*colour online*]. Map of Sweden. The two most northern counties of Sweden (Norrbotten and Västerbotten) are shown in darker colour.

In total, 2·9% of the population sampled during 2009 in the two northernmost counties of Sweden (Fig. 1) were positive for SINV antibodies. Of men, 3·7% were seropositive for SINV and for women 2·0% (*P* = 0·047) (Table 1). SINV antibodies were uncommon in the youngest age group but a similar prevalence was found across those aged >34 years (*P* for linear trend 0·3 and 0·6, for men and women, respectively). The specificity of the ELISA positive samples was verified by HI with titres ranging from 1:10 to 1:2560. There was an association between high HI titre and high ELISA titre (data not shown).

**Table 1.** Distribution of seropositivity for SINV according to sex and age

	Negative		Positive	
	*n*	%	*n*	%
Men, age group (yr)				
25–34	122	100	0	0·0
35–44	153	95·6	7	4·4
45–54	151	95·0	8	5·0
55–64	175	95·1	9	4·9
65–74	187	96·9	6	3·1
Women, age group (yr)				
25–34	127	99·2	1	0·8
35–44	163	97·6	4	2·4
45–54	153	98·1	3	1·9
55–64	166	97·1	5	2·9
65–74	168	98·2	3	1·8

Seropositivity for SINV was more common in subjects living in small and rural communities (*P* < 0·001) and with lower education (*P* = 0·013) (Table 2). Subjects owning a farm or working in agriculture did not differ from the population at large. In a stepwise logistic regression only rural living and low educational level predicted seropositivity, taking age, sex and smoking into account. Living in a rural area had an odds ratio of 4·3 (95% CI 2·0–9·2, *P* < 0·001) and living in municipalities an odds ratio 3·4 (95% CI 1·6–7·3, *P* = 0·002) compared to living in the city. Higher education halved the risk (OR 0·42, 95% CI 0·17–1·01, *P* = 0·054). Age and sex were not significant predictors.

**Table 2.** Prevalence of seropositivity to SINV according to demography, lifestyle and prevalent disease

		Positive for SINV (%)	*P*
Size of residential area	>15 000	1·3	<0·001
	1000–15 000	4·4	
	<1000	5·8	
Level of education	No university studies	3·5	0·013
	University studies	1·3	
Daily smoker	Yes	4·6	0·14
	No	2·6	
Previous myocardial infarction	Yes	2·5	0·9
	No	2·9	
Previous stroke	Yes	9·3	0·011
	No	2·7	
Diabetes	Yes	2·6	0·9
	No	3	
Treatment for hypertension	Yes	3·2	0·6
	No	2·7	
Lipid-lowering drugs	Yes	3·0	0·9
	No	2·9	
Myalgia	Yes	3·8	0·6
	No	2·8	
Arthralgia	Yes	4·5	0·3
	No	2·7	
Calf pain	Yes	6·3	0·011
	No	2·6	
Steroid treatment	Yes	2·1	0·6
	No	2·9	
Small farm	Yes	6·2	0·2
	No	2·8	
Large farm	Yes	0	0·7
	No	2·9	
Agricultural work	Yes	5·6	0·3
	No	2·8	

There were no differences in presence of SINV IgG antibodies according to smoking, previous myocardial infarction or diabetes. Of the participants, 4·7% were treated by a physician for myalgia and 6·7% for arthralgia. They did not differ in SINV seropositivity from those not treated. Twenty per cent of the subjects were treated for hypertension but did not differ in seropositivity from subjects without treatment. Calf pain on exertion was reported by 9% and here, SINV seropositivity was more common than in those without calf pain (*P* = 0*·*011).

Seropositivity was associated with somewhat higher waist circumference (*P* = 0·1) and higher diastolic blood pressure (*P* = 0·037), a difference of 3·0 mmHg (95% CI 2·0–6·0) (Table 3). Age, body mass index, systolic blood pressure, cholesterol or creatinine levels did not differ (*P*> 0·1) (Table 3).

**Table 3.** Risk factors associated with presence of SINV IgG in northern Sweden

	SINV serology		
	Negative	Positive	
	Mean	Mean	*P*
Age (years)	50·9	53·5	0·14
Body mass index (kg/m^2^)	26·8	27·6	0·2
Waist circumference (cm)	89·8	93·0	0·1
Hip circumference (cm)	101	103	0·2
Diastolic blood pressure (mmHg)	79	82	0·037
Systolic blood pressure (mmHg)	127	128	0·7
Total cholesterol (mmol/l)	5·7	5·8	0·9
Creatinine	85	87	0·3

Subjects who reported a previous stroke (*n* = 49, *n* = 46 with valid serology) were more often seropositive (9·3%) compared to those without a stroke (2·7%) (*P* = 0·011) (Table 2). In a logistic stepwise regression, only seropositivity for SINV and age were independent predictors of the risk for stroke (OR 4·3, 95% CI 1·4–13·0, *P* = 0·011). Sex, rural living, education, smoking, waist circumference, diastolic blood pressure and smoking were not independent predictors.

## DISCUSSION

The SINV seroprevalence of 2·9% suggested an increase in SINV infection during the last 25 years in this part of Sweden. In a Swedish study performed on human sera collected during 1981–1987 the SINV antibody prevalence showed a geographical distribution with the highest prevalence in central Sweden (3·6%) and decreasing prevalence both north and south of this region [8]. In the 1980s only 0·1% of the population in Norrbotten, the northernmost county in Sweden, were SINV antibody positive [8], compared to 2·9% in our study. It can be speculated that exposure to SINV has been more common during the last 25 years and there is data from Finland supporting a continuous increase in the incidence of SINV in northern Europe. The increase in Finland has been evident since the 1970s and is suggested to result from introduction of SINV into northern Europe during the 1960s to 1970s, most probably by SINV-infected migrating birds [2, 17]. This could also be the case for Sweden and perhaps other countries in Europe. For that reason an increased surveillance would be of importance and mandatory reporting of SINV infection should be considered.

In our study SINV antibodies were uncommon in young persons, but with no clear age dependence in older groups, suggesting a recent introduction, after the 1980s, and increase in this region. The antibody analysis methods we used were different from previous studies and we cannot exclude that part of the difference in seropositivity could depend on a more sensitive method.

Rural living, lower education and male sex were associated with higher prevalence. In a recently published population-based case-control study in Finland exposure to mosquito bites and spending time in woods or marshland were independently associated with SINV infection which may suggest more exposure to mosquito bites in men in rural areas [2, 22].

There was no association between SINV seropositivity and arthralgia and myalgia in our study. It should be borne in mind that these symptoms are prevalent in the population with 5–7% being treated. We note that seropositive subjects had more abdominal obesity and higher diastolic blood pressure, risk factors that may relate to lower socioeconomic status, indicating a higher cardiovascular risk. There were no differences in myocardial infarction, but the prevalence of previous stroke, based on 46 cases, was markedly increased even after adjustment for possible confounders. Moreover, there were more SINV antibody positives that had experienced calf pain on exertion, which could be a corroborative indication for arteriosclerotic disease. In the large INTERSTROKE study hypertension and abdominal obesity were among the most important risk factors for stroke [23], underscoring a possible association between SINV and stroke. On the other hand, this may be a spurious observation due to residual confounding from socioeconomic status and rural living. This association between presence of SINV antibodies and stroke was an unexpected finding. However, SINV can cause long-term (or permanent) arthritic/arthralgic disease and it is possible that low-grade chronic inflammation may increase vulnerability for stroke, as has recently been reported for inflammatory arthritis [24] and rheumatoid arthritis in the northern Sweden MONICA Study [25]. A systematic review and meta-analysis noted a doubled risk for stroke in patients with rheumatoid arthritis [26]. There is growing support for a causal role for infection in triggering stroke, linking infection and inflammation, thrombosis and stroke [27, 28]. Furthermore, remarkable similarities have been found between pro-inflammatory macrophage responses induced by SINV infection and those observed in rheumatoid arthritis, despite the different aetiologies of infectious and autoimmune arthritides [29]. Interestingly, the genetic predisposition for SINV infection, HLA-DRB1*01, has also been linked to rheumatoid arthritis and similar genetic predisposing factors may contribute to the development of SINV-induced and autoimmune arthritides resulting in disease with similar features although different aetiologies are involved [3]. Inflammation after infection in the brain could also play a part and although SINV has never been associated with neuroinvasion in humans, SINV causes encephalomyelitis in mice and the alphaviruses RRV and CHIKV could be neuroinvasive and cause neurological disease in humans [30]. Furthermore, the closely related alphaviruses Western equine, Eastern equine, and Venezuelan equine encephalitis viruses all cause neurological disease in humans [30].

In conclusion, the incidence of SINV infection has increased in northern Sweden and previous infection was associated with higher diastolic blood pressure, low education level, living in rural areas and independently associated with stroke. The signs of emergence of SINV in northern Sweden warrant further studies on the ecology and vector biology of SINV including the role of migratory and resident birds. Furthermore, compared to the considerable seroprevalence, relatively few cases are clinically recognized which indicates that SINV infection is a neglected disease in Sweden as well as in many other European countries.

## Supplementary Material

Supplementary Material Supplementary information supplied by authors.

## Supplementary material

For supplementary material accompanying this paper visit https://doi.org/10.1017/S0950268813002239. click here to view supplementary material
