# Supplementary material for: Seroprevalence of Sindbis virus and associated risk factors in northern Sweden
Source: Epidemiol Infect. 2013 Sep 13;142(7):1559–65. doi: 10.1017/S0950268813002239 (PMC9167656; doi:10.1017/S0950268813002239)
Supplement: Supplementary Material — Supplementary information supplied by authors. [file S0950268813002239sup001.doc]

**SUPPLEMENTARY ONLINE APPENDIX**

# Translation of questions from the 2009 MONICA population survey

(<http://www9.umu.se/phmed/medicin/monica/pdf/2009_1.pdf>)

Which is the highest education level you have achieved?

Do you presently smoke cigarettes regularly (one or more cigarette per day)?

Did you take antihypertensive drugs during the latest 2 weeks?

Do you have diabetes mellitus?

Have you been hospitalized for a confirmed myocardial infarction (clot in the heart)?

Have you had a stroke (bleeding in the brain or clot in the brain)? If yes, give the name of the hospital and the year

Are you being treated or checked for rheumatoid joint or muscle ailments in the form of pain/ache/stiffness

Do you frequently get pain in your calves when you walk uphill, in stairs or on level ground?

Which is your occupation /your employment?

*In order to identify possible occupational risks for SINV infection, occupations with similar assumed exposure to the vector were grouped together as; 1) office personnel; 2) teaching, clergy, police and military; 3) healthcare workers; 4) construction, wood industry and plumbers; 5) engineers; 6) transportation, trade and service; 7) industry; 8) agricultural work.*

Is your farm regarded as a small or a large farm (Only to be answered by farmers). How many acres of meadowland and forest are do you have?
